# Innovative Adsorbents for Pollutant Removal: Exploring the Latest Research and Applications

**DOI:** 10.3390/molecules29184317

**Published:** 2024-09-11

**Authors:** Muhammad Saeed Akhtar, Sajid Ali, Wajid Zaman

**Affiliations:** 1 Department of Chemistry, Yeungnam University, Gyeongsan 38541, Republic of Korea; msakhtar@yu.ac.kr; 2 Department of Horticulture and Life Science, Yeungnam University, Gyeongsan 38541, Republic of Korea; drsajid@yu.ac.kr; 3 Department of Life Sciences, Yeungnam University, Gyeongsan 38541, Republic of Korea

**Keywords:** adsorbents, contaminants, environmental protection, nanocellulose, metal–organic frameworks (MOFs), pollutant removal

## Abstract

The growing presence of diverse pollutants, including heavy metals, organic compounds, pharmaceuticals, and emerging contaminants, poses significant environmental and health risks. Traditional methods for pollutant removal often face limitations in efficiency, selectivity, and sustainability. This review provides a comprehensive analysis of recent advancements in innovative adsorbents designed to address these challenges. It explores a wide array of non-conventional adsorbent materials, such as nanocellulose, metal–organic frameworks (MOFs), graphene-based composites, and biochar, emphasizing their sources, structural characteristics, and unique adsorption mechanisms. The review discusses adsorption processes, including the basic principles, kinetics, isotherms, and the factors influencing adsorption efficiency. It highlights the superior performance of these materials in removing specific pollutants across various environmental settings. The practical applications of these adsorbents are further explored through case studies in industrial settings, pilot studies, and field trials, showcasing their real-world effectiveness. Additionally, the review critically examines the economic considerations, technical challenges, and environmental impacts associated with these adsorbents, offering a balanced perspective on their viability and sustainability. The conclusion emphasizes future research directions, focusing on the development of scalable production methods, enhanced material stability, and sustainable regeneration techniques. This comprehensive assessment underscores the transformative potential of innovative adsorbents in pollutant remediation and their critical role in advancing environmental protection.

## 1. Introduction

Over the past century, increased industrialization and urbanization have caused significant environmental pollution, especially in water bodies [1]. Contaminants such as heavy metals, dyes, pesticides, pharmaceuticals, and other organic pollutants are now frequently present in wastewater, posing significant hazards to human health and ecosystems [2,3]. Traditional pollutant removal processes, such as chemical precipitation, ion exchange, and membrane filtration, encounter challenges like high costs, energy requirements, and secondary pollutant generation. As a result, there is an increasing demand for more sustainable and efficient solutions [4,5]. Adsorption has gained attention as a feasible alternative due to its simplicity, cost-effectiveness, and ability to eliminate a variety of pollutants [6,7]. Recent research has demonstrated the potential of adsorption-based materials to efficiently remove pollutants, including heavy metals, pharmaceuticals, and other emerging contaminants from environmental media [8,9,10]. This process offers a sustainable and environmentally friendly alternative compared to traditional methods, as it avoids complex chemical reactions and can be applied across various environmental matrices such as water, soil, and air. Researchers are currently focused on developing new adsorbents that target specific contaminants effectively. These new materials are intended to be efficient, eco-friendly, and economically viable. Often derived from abundant natural resources or industrial byproducts, they align with the principles of green chemistry and the circular economy.

The potential of non-conventional adsorbents to outperform traditional ones in terms of capacity, selectivity, and reusability has increased their appeal for use in pollutant removal [11,12]. Materials such as nanocellulose, chitosan-based nanocomposites, and metal–organic frameworks (MOFs) demonstrated exceptional characteristics that render them well suited for environmental applications [13]. Modern adsorbents are produced through various advanced synthetic procedures that enhance their surface area, porosity, and functional groups, resulting in their exceptional ability to capture pollutants [14,15]. For instance, nanocellulose and cellulose-based hydrogels are produced from natural fibers and modified to boost their adsorption efficiency [16]. Cyclodextrin polymers, with their distinct cavity structures, can encapsulate organic molecules, while lignocellulosic resources and organic wastes provide a more sustainable and economically viable alternative to synthetic adsorbents [17]. Similarly, advanced materials like super-chalcogens, molecularly imprinted polymers, and metal–organic frameworks exhibit high selectivity towards specific pollutants, making them ideal for targeted removal applications [18,19,20].

These adsorbents can treat a wide range of contaminants, including heavy metals like lead and mercury, as well as organic pollutants like dyes, medicines, and pesticides [21]. Emerging contaminants like per- and polyfluoroalkyl substances (PFASs), microplastics, and endocrine-disrupting chemicals also fall within the scope of innovative adsorbent research [22,23,24]. These pollutants pose significant challenges due to their persistence, toxicity, and potential for bioaccumulation, necessitating the development of adsorbents capable of efficiently and selectively removing them from water and wastewater. The potential applications of these innovative adsorbents are not limited to conventional water and wastewater treatment. They have the potential to be employed in air purification systems, industrial effluent treatment, and groundwater remediation [18,23]. The versatility and adaptability of these materials make them suitable for various environmental contexts, offering a robust solution to pollution control. Moreover, the economic viability of these adsorbents is further enhanced by their capacity to regenerate and reuse, rendering them an appealing choice for large-scale applications.

This review offers an in-depth analysis of the latest developments in the area of cutting-edge adsorbents and their use in the removal of pollutants. The growing attention on these materials is driven by their distinct physicochemical properties, which enable high adsorption capacities and efficient removal of a variety of contaminants. Furthermore, many of these adsorbents can be regenerated and reused, reducing the overall cost and environmental footprint of the adsorption process. The primary objective of this review is to collect and evaluate the current state of research on innovative adsorbents that are being used for pollutant removal from water and wastewater. This includes an examination of different kinds of adsorbents, their synthesis methods, structural characteristics, and adsorption mechanisms. By providing a detailed understanding of these aspects, this review aims to highlight the strengths and limitations of various adsorbent materials and identify areas that require additional research. Additionally, this review evaluates the environmental impact, examines the economic feasibility, and investigates the practical applications of these adsorbents in real-world scenarios. Moreover, this review extends beyond existing comprehensive reviews by focusing on recent advances in innovative adsorbents, such as metal–organic frameworks (MOFs), nanocellulose-based materials, and biochar composites, highlighting their unique adsorption mechanisms and potential applications. In addition to detailing adsorption kinetics and mechanisms, this review provides specific insights into application-based adsorbent selection for distinct pollutants, such as wastewater treatment, air pollution control, and soil remediation. Finally, this review critically examines the economic and environmental challenges of implementing these adsorbents at scale, providing a practical perspective that is often overlooked in previous reviews. By identifying these challenges and potential solutions, this review aims to direct future research efforts toward the most promising and impactful areas in the field of environmental remediation.

## 2. Overview of Adsorption Processes

### 2.1. Basic Principles

**Figure 1:**
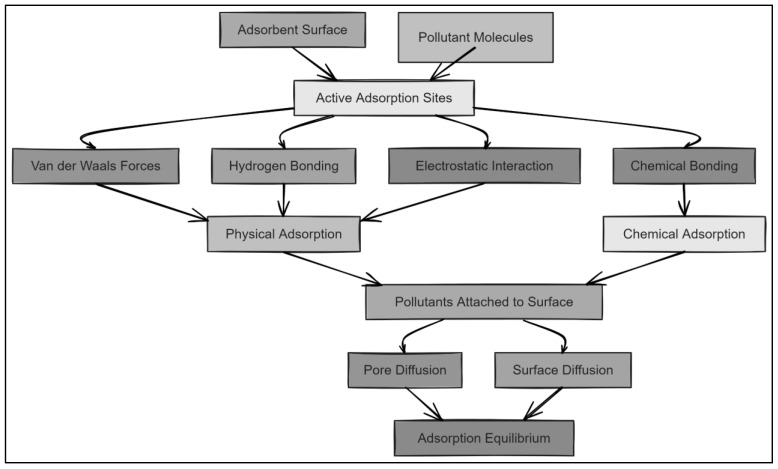
Mechanism of adsorption process.

Adsorption is a process in which molecules from a liquid or gas phase attach themselves to the surface of a solid adsorbent [25]. This process is predominantly driven by surface forces and can be classified into two types: physisorption and chemisorption. Physisorption relies on weak van der Waals forces, making it a reversible and relatively low-energy process (Figure 1). In contrast, chemisorption involves the formation of chemical bonds between the adsorbate and the adsorbent surface, leading to a stronger and often irreversible interaction [26]. The efficiency of adsorption is influenced by various factors, such as the nature of the adsorbate, the surface area and porosity of the adsorbent, and the operating conditions such as temperature and pressure. High surface area and porosity are critical for providing ample sites for adsorbate molecules to adhere to, thereby increasing the adsorption capacity [27,28].

Additionally, thermodynamic parameters such as enthalpy (ΔH), entropy (ΔS), and Gibbs free energy (ΔG) provide valuable information regarding the feasibility and spontaneity of the adsorption process. A negative Gibbs free energy (ΔG) indicates a spontaneous process, while the enthalpy change (ΔH) determines whether the process is endothermic or exothermic. These thermodynamic parameters are essential for understanding the energy changes during adsorption and help in optimizing adsorption systems for various environmental applications [29]. For instance, the adsorption of corrosion inhibitors on metal surfaces can be analyzed using thermodynamic parameters to assess the interaction strength and adsorption mechanism [30].

Density Functional Theory (DFT) simulations have emerged as an essential computational tool to provide deeper insights into adsorption mechanisms at the molecular level. DFT helps predict adsorption energies, charge transfer, and the interaction between adsorbates and the surface of adsorbents, which are crucial for rationalizing the adsorption behavior observed experimentally. Recent DFT studies have been employed to model the adsorption of different molecules onto substrate surfaces, offering valuable insights into the active sites, bonding interactions, and adsorption energies, which are difficult to capture through experimental methods alone [31,32]. For example, DFT can provide critical information on the adsorption mechanism of contaminants on advanced adsorbents, such as metal–organic frameworks (MOFs) or graphene-based materials [33].

The effectiveness of the adsorption process is also significantly influenced by the molecular size, polarity, and concentration of the adsorbate. The adsorption process can be characterized through various models and theories that assist in predicting the behavior of adsorbates on adsorbent surfaces [34,35]. Understanding these fundamental principles is critical for developing and optimizing adsorbents for specific applications. For example, adsorbents with functional groups designed to interact with specific contaminants can significantly enhance selectivity and efficiency. Moreover, advances in nanotechnology and material science have assisted the development of adsorbents with precisely controlled structures and functionalities, thereby facilitating the development of more effective pollutant removal technologies.

#### Mechanisms of Adsorption: Physisorption vs. Chemisorption

In the adsorption process, adsorbents can interact with pollutants through two primary mechanisms: physisorption and chemisorption. Understanding the difference between these mechanisms is critical for selecting the appropriate adsorbent for a given pollutant.

Physisorption involves weak van der Waals forces, making it a reversible process with lower adsorption energy. Adsorbents like activated carbon and nanocellulose predominantly rely on physisorption due to their high surface area and porous structure. These materials are particularly effective for adsorbing organic molecules and gases in environmental applications like air purification and the removal of dyes and volatile organic compounds.

Chemisorption, on the other hand, involves the formation of stronger chemical bonds (covalent or ionic) between the adsorbent and the adsorbate. Adsorbents such as metal–organic frameworks (MOFs), zeolites, and modified clays often operate through chemisorption, which provides a higher degree of specificity and irreversibility. These materials are highly suitable for targeting heavy metals and other inorganic pollutants, as they form stable complexes with metal ions.

### 2.2. Adsorption Isotherms

Adsorption isotherms are graphical representations of how adsorbates interact with adsorbents at constant temperature. They are crucial for understanding the adsorption capacity and mechanism [36]. Langmuir and Freundlich are the most frequently used isotherms. The Langmuir isotherm describes monolayer adsorption on a homogeneous surface with a finite number of identical sites, while the Freundlich isotherm describes adsorption on heterogeneous surfaces with varying affinities [37]. The Langmuir isotherm is particularly effective for describing adsorption processes in which saturation of the adsorbent surface occurs, which indicates a maximal adsorption capacity [38]. In contrast, the Freundlich isotherm is more appropriate for adsorption on heterogeneous surfaces and does not predict saturation, indicating the varying affinities and capacities prevalent on the adsorbent surface [37,39].

It is essential to comprehend these isotherms to design and choose the best adsorbents for various contaminants. By fitting experimental data to these models, researchers can gain deeper insights into the adsorption mechanisms and efficiency of various adsorbents. This knowledge facilitates the prediction of the performance of adsorbents under diverse conditions and improves them for specific applications, hence increasing their practical utility in pollutant removal [39].

### 2.3. Kinetics of Adsorption

The kinetics of adsorption describe the rate at which adsorbates are removed from the liquid or gas phase and adhere to the adsorbent surface. Understanding adsorption kinetics is crucial for assessing the practicality of adsorption processes in real-world applications. Kinetic studies typically examine factors such as contact time, initial adsorbate concentration, and temperature [40,41]. Two prevalent models used to describe adsorption kinetics are the pseudo-first-order and pseudo-second-order models. These models are essential for understanding the rate at which equilibrium is achieved and the overall dynamics of the adsorption process. They also provide insights into the adsorption mechanisms, whether they are driven by physical or chemical interactions, and highlight potential limitations in practical applications [42].

1. Pseudo-first-order kinetics: This model assumes that the rate of adsorption is proportional to the number of available adsorption sites. It is represented by the following equation:

(1) $$\log\left(q_{e}-q_{t}\right)=\log q_{e}-\frac{k_{1}}{2.303}t$$

2. Pseudo-second-order kinetics: This model assumes that adsorption follows chemisorption, where the rate of occupation of adsorption sites is proportional to the square of the number of unoccupied sites. The equation is as follows:

(2) $$t/q_{t}=\left(1/k_{2}*q_{e}^{2}\right)+\left(t/q_{e}\right)$$

*q_e_* is the amount of adsorbate adsorbed at equilibrium (mg/g).

*q_t_* is the amount of adsorbate adsorbed at time *t* (mg/g).

*k*_1_ is the rate constant of the pseudo-first-order adsorption (1/min).

*k*_2_ is the rate constant of the pseudo-second-order adsorption (g/mg·min).

Recently, a study on sustainable cellulose derivatives applied the pseudo-second-order model, indicating chemisorption as the dominant mechanism [43]. This aligns with other bio-based adsorbents, such as biochar and nanocellulose, and supports the development of more sustainable and efficient adsorbents for pollutant removal. Other important kinetic factors include the diffusion of adsorbate molecules within the pores of the adsorbent and the interaction energies involved. These considerations are essential for optimizing the contact time and enhancing the overall efficiency of the adsorption process [44,45]. By analyzing adsorption kinetics, researchers can determine the optimal conditions for maximum adsorption efficiency and develop strategies to improve the performance of adsorbents in real-world applications.

### 2.4. Factors Affecting Adsorption Efficiency

The efficiency of the adsorption process is influenced by several factors, including the properties of the adsorbent, the characteristics of the adsorbate, and the environmental conditions. The adsorption capacity and selectivity of the adsorbent are significantly influenced by its surface area, porosity, and functional groups [46,47]. Adsorbents with high surface area and well-developed porosity can offer more active sites for adsorption, thereby enhancing efficiency [48]. The characteristics of the adsorbate, such as molecular size, polarity, and concentration, significantly impact the adsorption process. Smaller molecules can more readily diffuse into the pores of the adsorbent, while polar molecules tend to have stronger interactions with adsorbents that possess polar or charged functional groups. The initial concentration of the adsorbate in the solution influences the driving force for mass transfer, with higher concentrations generally resulting in increased adsorption rates [48,49].

Environmental factors, including pH, temperature, and ionic strength, significantly influence adsorption efficiency. The pH of the solution can impact the ionization state of both the adsorbate and the adsorbent, thereby affecting the adsorption capacity. For example, acidic conditions can enhance the adsorption of cationic species, whereas basic conditions may favor the adsorption of anionic species [50]. Temperature influences the kinetic energy of adsorbate molecules as well as the interactions between the adsorbate and adsorbent. Mostly, adsorption capacity increases with temperature in chemisorption processes, whereas in physisorption, it may decrease due to higher desorption rates [51,52].

Ionic strength, determined by the presence of other ions in the solution, can impact the adsorption process through competitive adsorption and changes in the electric double layer at the adsorbent surface. High ionic strength can decrease the adsorption of ionic species by shielding the charged sites on the adsorbent or by competing for adsorption sites [53,54]. A thorough understanding of these factors is crucial for optimizing the adsorption process and developing more efficient and selective adsorbents for pollutant removal. Understanding the basic principles, adsorption isotherms, kinetics, and factors affecting adsorption efficiency is essential for designing and optimizing innovative adsorbents for pollutant removal. These insights lay the groundwork for developing materials that are not only effective in contaminant removal but also economically feasible and environmentally sustainable.

## 3. Innovative Non-Conventional Adsorbents

**Figure 2:**
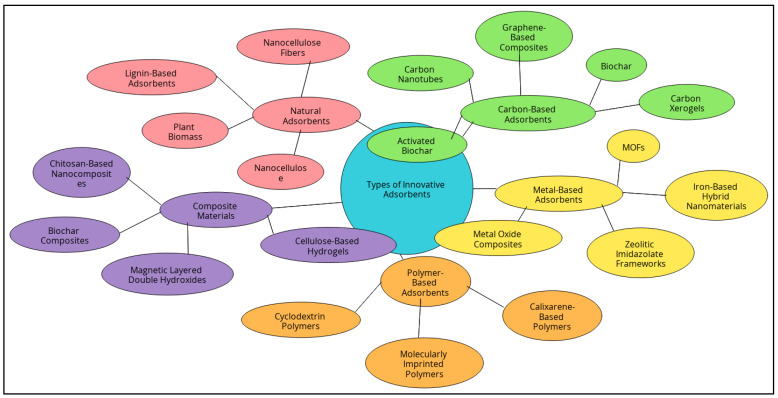
Overview of innovative adsorbent types.

Recent advancements in adsorbent materials have led to the development of innovative non-conventional adsorbents designed to tackle environmental pollution more effectively and sustainably. These materials, which include naturally derived substances like nanocellulose and biochar as well as synthetically engineered options like metal–organic frameworks (MOFs) and molecularly imprinted polymers (MIPs), offer significant improvements over traditional adsorbents. They are characterized by high adsorption capacities, selectivity for specific pollutants, and the ability to be regenerated and reused (Figure 2). To better understand the capabilities of these adsorbents, Table 1 summarizes the primary mechanisms, target pollutants, and their use in various applications.

### 3.1. Three-Dimensional Graphene-Based Adsorbents

Advanced materials that combine the exceptional properties of graphene with a three-dimensional structure are known as three-dimensional (3D) graphene-based adsorbents [55]. These materials offer high surface area, mechanical strength, and tunable porosity. They are produced by constructing graphene sheets into a 3D network using methods such as freeze-drying, hydrothermal treatment, or chemical vapor deposition [56]. The 3D structure of these adsorbents improves their adsorption capacity and kinetics. This structure facilitates the diffusion of pollutants and provides more accessible active sites. The graphene surface has a high affinity for specific contaminants, which is further enhanced by functionalizing the surface with various groups such as carboxyl, hydroxyl, and amine. Three-dimensional graphene-based adsorbents have shown high efficiency in removing heavy metals, dyes, pharmaceuticals, and other organic pollutants from water [55].

The potential of 3D graphene-based adsorbents in environmental remediation applications has been demonstrated through research, which has shown that they can achieve high removal efficiencies and swift pollutant uptake. Additionally, their mechanical strength and stability facilitate regeneration and recurrent use. The primary objectives of ongoing research are to enhance the functionalization processes, develop scalable synthesis methods, and investigate novel applications for these advanced adsorbents.

### 3.2. Adsorbents from Stainless Steel Slag

Stainless steel slag, a byproduct of stainless steel production, is a valuable resource that can be repurposed to create high-performance adsorbents for environmental remediation and waste management. With its abundance of metal oxides and compounds, stainless steel slag can be transformed through various activation and modification processes to efficiently remove pollutants. This presents a cost-effective and sustainable solution for addressing environmental challenges [57]. The process of turning stainless steel slag into adsorbents involves adding functional groups, heating it up, and activating it with chemicals. These treatments enhance the surface area, porosity, and adsorption capacity of the resulting materials. Adsorbents made from stainless steel slag have shown great effectiveness in removing heavy metals, organic contaminants, and nutrients from both wastewater and water.

Research has shown that stainless steel slag can be effectively and affordably used as an adsorbent in environmental applications. Using industrial byproducts to create adsorbents aligns with the principles of sustainable development and the circular economy. Ongoing research focuses on understanding the adsorption mechanisms, refining the conversion processes, and evaluating the long-term performance of the adsorbents.

### 3.3. Biochar Composites

Biochar is a carbon-rich material produced through the pyrolysis of biomass. It has shown great promise as an adsorbent for removing pollutants. Due to its porous structure, large surface area, and high cation exchange capacity, biochar is effective in adsorbing a wide range of contaminants, including heavy metals, organic pollutants, and nutrients [58]. Biochar composites, which combine biochar with other materials, offer enhanced adsorption properties and broader applicability. The process of creating biochar composites involves incorporating materials such as metal oxides, clays, and polymers into the biochar structure. This results in combined effects that enhance the biochar’s capacity to adsorb and selectively capture substances. By adjusting the pyrolysis conditions and choosing the appropriate additives, these composites can be tailored to effectively target specific contaminants. For example, biochar–metal oxide composites exhibit improved ability to attract and retain heavy metals, while biochar–clay composites are effective at removing organic pollutants [59].

Numerous studies have demonstrated the effectiveness of biochar composites in various environmental applications, including soil restoration, wastewater purification, and air cleansing. The use of agricultural and forestry residues as raw materials for biochar production aligns with sustainable development goals, providing a cost-effective and environmentally friendly method for eliminating pollutants. Ongoing research focuses on enhancing synthesis methods, understanding adsorption mechanisms, and evaluating the durability of biochar composites.

### 3.4. Calixarene-Based Polymers

Calixarenes are cyclic oligomers with a basket-like structure that can encapsulate a variety of molecules within their hydrophobic cavities. Calixarene-based polymers are synthesized by polymerizing calixarene units or grafting them onto various support materials, resulting in adsorbents with high selectivity and adsorption capacity for specific pollutants [60].

The unique structure of calixarenes allows them to selectively bind to certain contaminants based on the size and shape of their cavities. This selectivity is particularly useful for targeting organic pollutants, such as pesticides, pharmaceuticals, and endocrine-disrupting chemicals, which can fit into the calixarene cavities [61].

Calixarene-based polymers have been shown to effectively remove a wide range of organic pollutants from water. The adsorption mechanisms involve host–guest interactions, where the pollutants are encapsulated within the calixarene cavities. The high selectivity, combined with the ease of regeneration, makes calixarene-based polymers a promising class of adsorbents for environmental remediation. Ongoing research focuses on developing new calixarene derivatives and composites to expand their range of applications and improve their performance in complex water matrices [62].

### 3.5. Carbon Nanotubes

Carbon nanotubes (CNTs) are cylindrical nanostructures made of rolled-up graphene sheets. They possess exceptional qualities such as high surface area, mechanical strength, and electrical conductivity. CNTs can be classified into two types: single-walled carbon nanotubes (SWCNTs) and multi-walled carbon nanotubes (MWCNTs), each with distinct structural and adsorption properties [63]. CNTs have been extensively studied for their ability to adsorb various contaminants, such as heavy metals, organic chemicals, and gases. Their large surface area and the presence of π-electrons enable interactions with different pollutants, making them highly effective adsorbents. The adsorption ability and selectivity of CNTs can be enhanced by functionalizing them with carboxyl, hydroxyl, and amine groups [64]. CNTs have shown potential for use in treating water and effluent, air purification, and soil remediation, as demonstrated by research. Furthermore, the effectiveness of CNTs has been improved through the development of composite materials that include metal oxides, polymers, or other adsorbents [65]. The primary goal of current research is to address the challenges related to environmental impact, cost, and scalability of CNT synthesis and application.

### 3.6. Carbon Xerogels

Carbon xerogels are highly porous carbon materials synthesized through the sol–gel process, followed by pyrolysis and dehydration. This material is ideal for a variety of environmental applications due to its high surface area, customizable porosity, and exceptional adsorption properties. The sol–gel process empowers the development of materials specifically designed for precise adsorption duties by enabling precise control over the pore structure and surface chemistry of the xerogels [66]. The process of synthesizing carbon xerogels involves polymerizing precursors such as formaldehyde and resorcinol in the presence of a catalyst. The resulting gel is then dried and pyrolyzed to produce the final carbon xerogel. The adsorption capacity and selectivity of the xerogel can be enhanced by functionalizing the surface with groups like carboxyl, hydroxyl, and amine. Research has illustrated the remarkable capability of carbon xerogels to effectively eliminate a diverse range of pollutants, such as organic compounds, heavy metals, and radionuclides, from both wastewater and water. Their extraordinary ability to rapidly adsorb pollutants and their substantial pollutant absorption are attributed to their adjustable porosity and expansive surface area [67]. Current research endeavors are primarily focused on refining synthesis processes, exploring innovative functionalization techniques, and meticulously scrutinizing the enduring performance of carbon xerogels in various environmental applications.

### 3.7. Carbonaceous Waste from Oil Refineries

Petroleum coke and spent catalysts, which are carbonaceous refuse from oil refineries, are valuable resources for the production of adsorbents. There are numerous activation and modification procedures that can be employed to transform these materials into high-performance adsorbents, as they are typically rich in carbon. In addition to addressing the issue of industrial waste disposal, the utilization of carbonaceous waste offers a cost-effective solution for pollutant removal [68]. The process of converting carbonaceous detritus into adsorbents entails the impregnation of functional groups, thermal activation, and chemical activation. The surface area, porosity, and adsorption capacity of the resultant materials are all improved by these treatments. For instance, activated petroleum coke has demonstrated exceptional adsorption capabilities for organic pollutants and heavy metals, while spent catalysts can be tailored to adsorb specific contaminants [69].

Research has shown that carbonaceous waste-derived adsorbents are effective in removing various pollutants from water and wastewater. Using industrial byproducts to produce adsorbents aligns with the principles of circular economy and sustainable development. Current research efforts are focused on optimizing conversion processes, understanding adsorption mechanisms, and evaluating the long-term performance of these adsorbents.

### 3.8. Carbon-Based Aerogels from Waste Paper

An innovative solution for removing pollutants that combines high performance with sustainability involves using carbon-based aerogels derived from waste paper. These aerogels are created by pyrolyzing waste paper, resulting in a lightweight, highly porous material with a large surface area [56]. Besides addressing the problem of paper waste disposal, using waste paper as a raw material provides an environmentally beneficial way to produce adsorbents. The process of creating carbon-based aerogels involves converting waste paper into a carbon-rich material through hydrothermal treatment, followed by high-temperature pyrolysis. The resulting aerogels demonstrate excellent ability to adsorb a wide range of pollutants, including organic compounds, dyes, and heavy metals. This is because of the aerogels’ porous structure, which allows contaminants to diffuse into the material and be adsorbed onto its surface [70].

Studies have shown that carbon-based aerogels made from waste paper can rapidly adsorb pollutants and achieve high removal rates. Their high porosity and low density make them suitable for various environmental applications, including soil remediation, air purification, and water and wastewater treatment. Current research focuses on optimizing the synthesis processes, exploring new applications, and understanding the adsorption mechanisms of these aerogels.

### 3.9. Cellulose-Based Hydrogels

Cellulose-based hydrogels represent another class of innovative adsorbents that have gained significant attention for pollutant removal [71]. These hydrogels are hydrophilic, three-dimensional polymer networks that can swell in water and retain a large amount of water within their structure. The incorporation of cellulose into hydrogels improves their mechanical strength, biocompatibility, and adsorption capacity, making them ideal for environmental applications [72,73].

The synthesis of cellulose-based hydrogels involves the crosslinking of cellulose or its derivatives with various polymeric materials. This crosslinking can be achieved through physical or chemical methods, resulting in hydrogels with tailored properties for specific adsorption tasks. For example, incorporating functional groups such as carboxylates or amines into the hydrogel matrix can significantly enhance its affinity for heavy metals and organic pollutants [16,73]. These hydrogels have proven effective in removing various contaminants from water including dyes, heavy metals, and pharmaceuticals. The high water retention capacity of hydrogels facilitates the diffusion of pollutants into the polymer network, where they are adsorbed onto the functional sites. Additionally, the reusability and easy regeneration of cellulose-based hydrogels make them a cost-effective and sustainable option for large-scale pollutant removal applications.

### 3.10. Chitosan-Based Nanocomposites

Chitosan, a biopolymer derived from chitin, is another innovative adsorbent that has been extensively studied for pollutant removal. Chitosan is composed of glucosamine and N-acetylglucosamine units, providing a high density of amino and hydroxyl groups that can be modified to enhance adsorption properties. Chitosan-based nanocomposites, which incorporate nanoparticles or other materials, offer improved adsorption performance due to their increased surface area and functional group availability [74].

The synthesis of chitosan-based nanocomposites involves the incorporation of various nanoparticles, such as metal oxides, carbon nanotubes, and graphene, into the chitosan matrix. These nanocomposites exhibit synergistic effects, combining the high adsorption capacity of chitosan with the unique properties of the nanoparticles, such as enhanced surface area and specific interactions with pollutants [75].

Chitosan-based nanocomposites have demonstrated high efficiency in removing a wide range of contaminants, including heavy metals, dyes, and pharmaceuticals. The adsorption mechanisms involve electrostatic interactions, chelation, and hydrogen bonding, facilitated by the functional groups on the chitosan and the nanoparticles [76]. The ability to regenerate and reuse these nanocomposites further enhances their practical applicability in water and wastewater treatment.

### 3.11. Cyclodextrin Polymers

Cyclodextrin polymers are another advanced class of adsorbents known for their unique ability to form inclusion complexes with various organic molecules. Cyclodextrins are cyclic oligosaccharides consisting of glucose units linked by α-1,4-glycosidic bonds, creating a toroidal structure with a hydrophobic interior and a hydrophilic exterior. This feature enables cyclodextrins to encapsulate hydrophobic organic pollutants within their cavities [77,78]. To enhance the practical pertinence of cyclodextrins in pollutant removal, they are frequently grafted onto different support materials, making cyclodextrin polymers with improved stability and adsorption capacity. These polymers are very effective in capturing and removing organic contaminants such as pesticides, pharmaceuticals, and endocrine-disrupting chemicals from water [12,50].

The strength of cyclodextrin polymers lies in their ability to selectively bind specific pollutants based on the size and shape of their hydrophobic cavities [79]. This selectivity, along with their high adsorption capacity and ease of regeneration, makes cyclodextrin polymers very effective for environmental remediation [17,80]. Moreover, ongoing research focuses on developing new cyclodextrin derivatives and composites to expand their range of applications and improve their performance in complex water matrices.

### 3.12. Geopolymers (Inorganic Polymers)

Geopolymers are formed by chemically activating aluminosilicate minerals such as fly ash, metakaolin, and slag using alkali substances. They are inorganic polymers with a three-dimensional network structure, providing excellent mechanical strength, thermal stability, and chemical resistance. Geopolymers have attracted attention as adsorbents due to their eco-friendliness, cost-efficiency, and ability to incorporate different functional groups to enhance pollutant removal [81]. Geopolymer synthesis involves mixing aluminosilicate precursors with alkaline solutions, like sodium hydroxide or potassium hydroxide, to create a solid structure. This process can be adjusted to produce geopolymers with specific porosities and surface traits suitable for adsorption. Geopolymers have a porous structure and reactive sites on their surfaces, giving them a high capacity for absorbing heavy metals, dyes, and organic contaminants [82].

Research has demonstrated that geopolymers can efficiently eliminate contaminants from water and wastewater, offering a sustainable and cost-effective substitute for traditional adsorbents. The utilization of industrial byproducts in geopolymer production is in line with the concept of a circular economy and waste valorization. Current research endeavors to improve the adsorption capabilities of geopolymers through modifications and composite formation, as well as to investigate their long-term stability and reusability.

### 3.13. Graphene-Based Composites

Graphene-based composites are advanced materials that combine graphene or graphene oxide with other substances to enhance their ability to adsorb. Graphene consists of a single layer of carbon atoms arranged in a hexagonal lattice. It has outstanding properties such as a large surface area, strong mechanical strength, and excellent electrical conductivity. Graphene and its derivatives have properties that make them highly effective for applications involving the removal of pollutants [83]. The process of synthesizing graphene-based composites involves combining graphene sheets with substances like metal oxides, polymers, and carbon nanotubes. These composite materials exhibit improved ability to attract and retain various pollutants such as heavy metals, dyes, and medicines due to their increased adsorption capabilities and selectivity. The presence of functional groups on graphene oxide such as hydroxyl, carboxyl, and epoxy groups enhances the adsorption process through different mechanisms including electrostatic interactions, π-π stacking, and hydrogen bonding [84].

Research has shown that composites using graphene can effectively remove pollutants from water and wastewater due to their high removal efficiency and quick adsorption kinetics. These composites are suitable for recycling and reusing, making them even more practical. Current research is focused on creating scalable synthesis technologies, exploring new composite materials, and understanding the molecular-level processes involved in removing pollutants.

### 3.14. Hydroxyapatite Nanoparticles

Hydroxyapatite (HAP) is a naturally occurring mineral form of calcium apatite. It is notable for its strong attraction to heavy metals and other harmful substances. HAP nanoparticles have been identified as highly effective adsorbents for environmental applications as a result of their high surface area and reactivity [85]. Precipitation, sol–gel, and hydrothermal processes are among the techniques that can be used to synthesize these nanoparticles. The adsorption capabilities of hydroxyapatite (HAP) nanoparticles can be related to their surface functional groups. These functional groups can interact with pollutants through mechanisms such as ion exchange, complexation, and precipitation. HAP nanoparticles exhibit exceptional efficacy in the removal of heavy metals, including lead, cadmium, and arsenic, from water. In addition, they have the ability to adsorb fluoride, phosphates, and organic contaminants, which makes them highly adaptable for water and wastewater treatment [86].

Studies have demonstrated that HAP nanoparticles may effectively achieve high rates of removing substances and have fast rates of adsorption. Their compatibility with living organisms and few negative effects on the environment further increase their attractiveness for practical use. The enhancement of composite materials by integrating HAP nanoparticles with other adsorbents is an ongoing process that aims to optimize their efficiency and broaden their scope in the field of pollutant removal.

### 3.15. Industrial Sludge

Industrial sludge, a byproduct of a variety of industrial processes, is composed of a combination of inorganic and organic compounds that can be employed as adsorbents to remove pollutants. The conversion of waste into valuable resources is a cost-effective solution for waste disposal, and the use of industrial wastewater as an adsorbent contributes to sustainable environmental management [87]. The process of converting industrial waste into adsorbents involves impregnating functional groups, thermal activation, and chemical activation. These treatments improve the surface area, porosity, and adsorption capacity of the resultant materials. Adsorbents derived from industrial sludge have shown great effectiveness in removing heavy metals, organic contaminants, and nutrients from both wastewater and water [88].

The potential of utilizing industrial effluent as a low-cost and highly effective adsorbent for environmental applications has been convincingly demonstrated through extensive research. Embracing the principles of circular economy and sustainable development, the utilization of industrial byproducts in the production of adsorbents represents a significant step towards minimizing waste and maximizing resource efficiency [89]. Current research efforts are concentrated on fine-tuning conversion processes, unraveling the intricacies of adsorption mechanisms, and evaluating the long-term performance of these innovative adsorbents.

### 3.16. Ionic-Liquid-Enhanced Adsorbents

Ionic liquids (ILs) are salts that are in the liquid state at comparatively low temperatures and are typically composed of organic cations and inorganic or organic anions. ILs are particularly appealing for improving the performance of adsorbents due to their distinctive properties, including high thermal stability, low volatility, and tunable solubility. Ionic-liquid-enhanced adsorbents are materials that incorporate ILs into their structure to enhance adsorption capacity and selectivity [90]. Impregnating or grafting ILs onto a variety of support materials, including silica, carbon, and polymers, is a crucial process for synthesizing ionic-liquid-enhanced adsorbents. This advanced material efficiently adsorbs a diverse array of contaminants, such as organic compounds, dyes, and heavy metals. The presence of ILs significantly enhances the adsorption properties by facilitating interactions with the contaminants and providing additional binding sites.

Researchers have shown that adsorbents enhanced with ionic liquids can achieve rapid adsorption kinetics and high removal efficiencies, making them suitable for environmental applications. The versatility of ionic liquids is further improved by the ability to adjust their properties through the selection of different cation–anion combinations. Current studies are focusing on optimizing synthesis processes, exploring new ionic liquid formulations, and understanding the mechanisms of pollutant removal in these materials.

### 3.17. Iron-Based Hybrid Nanomaterials

Iron-based hybrid nanomaterials are formed by combining iron oxides with other substances, such as carbon nanotubes, graphene, and polymers. This combination results in adsorbents that have improved characteristics. These hybrid materials exploit the reactive and magnetic characteristics of iron oxides, while also benefiting from the stability, surface area, and possibility for functionalization offered by other materials [91]. The production of iron-based hybrid nanomaterials entails techniques such as co-precipitation, sol–gel, and hydrothermal procedures. These materials have a strong ability to attract and hold onto large amounts of heavy metals, dyes, and organic contaminants. Iron oxides enhance redox processes and enable magnetic separation, while the supplementary components contribute to stability and augment the number of adsorption sites [92]. Studies have shown that iron-based hybrid nanoparticles are highly successful in eliminating a diverse array of contaminants from water. Their susceptibility to magnetic field-induced separation and subsequent regeneration for multiple uses renders them particularly advantageous for environmental applications. Current research endeavors seek to optimize their efficiency by advancing the design of novel hybrid architectures and implementing innovative functionalization methods.

### 3.18. Iron-Rich Red Mud

Iron-rich red mud is a residual substance generated as a result of the aluminum industry’s Bayer process, which is used to purify bauxite ore. This waste material consists of a variety of oxides, mostly iron oxide, which is responsible for its distinctive red hue. Red mud has been investigated as an inexpensive adsorbent for removing pollutants, because of its significant iron content and extensive surface area [93]. The utilization of red mud in environmental restoration encompasses its application in both raw and modified forms. Chemical modifications, such as acid activation and thermal treatment, can improve the ability of red mud to adsorb substances by enlarging its surface area and incorporating more functional groups. These alterations enhance its ability to absorb heavy metals, dyes, and organic contaminants from water [94].

Studies have demonstrated that red mud, which is rich in iron, may efficiently eliminate pollutants like arsenic, lead, and cadmium from liquid solutions. Utilizing this industrial byproduct as an adsorbent offers an economical method for removing pollutants and simultaneously tackles the problem of red mud disposal [95]. Current research is centered around improving modification techniques and broadening the scope of contaminants that can be efficiently eliminated with red mud.

### 3.19. Lignin-Based Adsorbents

Lignin, a primary component of lignocellulosic biomass, has garnered attention as a potential adsorbent for pollutant removal. This complex aromatic polymer is rich in functional groups, including phenolic and carboxylic groups, which contribute to its adsorption capacity. Its unique structure, with a high degree of polymerization and crosslinking, provides a robust framework for capturing pollutants [96,97].

The development of lignin-based adsorbents involves various chemical modifications to enhance their adsorption properties. Sulfonation, amination, and oxidation are common techniques used to introduce additional functional groups that improve the affinity for specific contaminants [97]. Lignin-based materials can be used in their natural form or as composites with other adsorbents, such as activated carbon or metal oxides, to improve their performance.

Research has shown the effectiveness of lignin-based adsorbents in removing heavy metals, dyes, and organic pollutants from water. The adsorption mechanisms involve complexation, ion exchange, and π-π interactions between the pollutants and the functional groups on the lignin structure. The abundance and low cost of lignin, combined with its high adsorption capacity, make it a promising material for large-scale environmental applications.

### 3.20. Lignocellulosic Resources and Organic Wastes

Lignocellulosic resources and organic wastes provide a sustainable and cost-effective source of adsorbent materials for pollutant removal. These materials, derived from agricultural residues, forestry byproducts, and industrial wastes, are both abundant and renewable. The primary components of lignocellulosic biomass are cellulose, hemicellulose, and lignin, each contributing to its adsorption properties [98,99].

The use of lignocellulosic biomass as adsorbents involves various pretreatment and modification techniques to enhance their adsorption capacity and selectivity. Physical treatments like grinding and sieving increase the surface area, while chemical modifications introduce functional groups that improve affinity for specific pollutants. Activated carbon, derived from lignocellulosic materials through processes like pyrolysis and chemical activation, has shown excellent performance in adsorbing a wide range of contaminants [99].

Studies have revealed the effectiveness of lignocellulosic adsorbents in removing heavy metals, dyes, and organic pollutants from aqueous solutions. The diverse functional groups present in these materials, such as hydroxyl, carboxyl, and phenolic groups, facilitate the adsorption process through various mechanisms, including ion exchange, complexation, and hydrogen bonding. The use of lignocellulosic biomass and organic wastes not only provides an efficient means of pollutant removal but also encourages the principles of waste valorization and circular economy.

### 3.21. Magnetic Layered Double Hydroxides

Layered double hydroxides (LDHs) are a type of material characterized by a distinctive layered structure, consisting of metal hydroxide layers that carry a positive charge and are separated by anions in the interlayer. Magnetic layered double hydroxides (LDHs), which comprise magnetic nanoparticles like iron oxide, have the additional benefit of easy separation from aqueous solutions by utilizing an external magnetic field. This characteristic renders them extremely appealing for real environmental applications [100]. Magnetic LDHs are synthesized using co-precipitation or hydrothermal processes, in which magnetic nanoparticles are integrated into the LDH structure. These materials have a strong ability to attract and hold heavy metals, dyes, and organic contaminants due to their vast surface area, adjustable gaps between layers, and functional groups on the surface [101].

The magnetic characteristics of these materials allow for effective separation and retrieval of the adsorbent following the removal of pollutants, hence decreasing operational expenses and enabling easy reuse. Studies have shown that magnetic LDHs are successful in many water treatment applications [100]. Ongoing research is focused on improving their stability, adsorption capacity, and selectivity by additional modifications and composite synthesis.

### 3.22. Mesoporous Silicas

Mesoporous silicas are a type of material that have structured pore architectures and large surface areas. The synthesis of these materials involves the use of surfactant templates, which result in the formation of uniform mesopores inside the silica matrix. These mesopores have diameters ranging from 2 to 50 nm. Mesoporous silicas possess a large surface area and flexible pore size, which makes them very suitable for adsorption purposes [102]. The adsorption properties of mesoporous silicas can be greatly improved by functionalizing them with different organic or inorganic groups. Affinity for certain contaminants can be enhanced by introducing functional groups such as amines, thiols, and carboxylates onto the surface of silica. This adaptability enables the creation of mesoporous silicas that are specifically designed to target specific pollutants [103].

Mesoporous silicas have attracted substantial research attention due to their capacity to eliminate heavy metals, dyes, and organic contaminants from water. Their large pore capacity allows simple diffusion of contaminants into the material, where they are adsorbed and attached to the functional sites. Mesoporous silicas are particularly efficient adsorbents due to their organized pore structure, which enables fast adsorption kinetics. Current research is dedicated to the development of novel synthesis processes and functionalization strategies to improve the effectiveness of mesoporous silicas in removing pollutants.

### 3.23. Metal Oxide Composites

Metal oxide composites are materials that utilize the combined features of multiple metal oxides to improve their adsorption effectiveness through synergistic effects. These composites can be produced by a range of methods, including sol–gel, co-precipitation, and hydrothermal approaches. Metal oxides, when combined, offer a substantial surface area, a diverse range of active sites, and enhanced stability, resulting in their exceptional efficacy for the removal of pollutants [104].

Titanium dioxide (TiO_2_), zinc oxide (ZnO), and iron oxide (Fe_2_O_3_) are often-utilized metal oxides in these composites. These materials have a strong ability to attract and hold a large number of contaminants, such as heavy metals, dyes, and organic chemicals. The inclusion of various metal oxides amplifies the adsorption mechanisms, including photocatalytic degradation, redox reactions, and ion exchange [105].

Studies have demonstrated that metal oxide composites can produce fast adsorption rates and significant levels of pollutant removal. Their capacity for regeneration and subsequent reuse significantly boosts their practical applicability. Advancements in synthesis techniques and the integration of supplementary functional groups are consistently enhancing the effectiveness and adaptability of metal oxide composites for environmental remediation purposes.

### 3.24. Metal–Organic Frameworks

Metal–organic frameworks (MOFs) are a type of crystalline material that consist of metal ions or clusters coordinated with organic ligands, resulting in the formation of porous structures. These materials have attracted considerable interest because of their remarkably large surface areas, tunable pore diameters, and the capacity to include customized functional groups for specific adsorption purposes. MOFs, due to their modular structure, offer a diverse range of possibilities for combining metals and ligands. This enables the creation of materials that possess specific adsorption properties [106]. Metal–organic frameworks (MOFs) demonstrate an exceptional ability to adsorb a wide variety of pollutants, such as heavy metals, volatile organic compounds (VOCs), and new contaminants such as pharmaceuticals and endocrine-disrupting chemicals. Their significant porosity enables fast adsorption kinetics and efficient absorption of pollutants, while the functional groups within the MOF structure offer specific binding sites for various contaminants. MOFs can be produced by solvothermal, hydrothermal, and microwave-assisted techniques, which enable accurate manipulation of their structural and chemical characteristics [106,107]. Studies have shown that MOFs have the ability to be highly effective in water and wastewater treatment applications. They can efficiently remove pollutants and quickly absorb them. Moreover, the practical usability of MOFs is improved by regenerating them through uncomplicated washing or thermal treatment. Current research is dedicated to the development of metal-organic frameworks (MOFs) that have improved stability and resistance to fouling. Additionally, researchers are investigating novel uses of MOFs in the field of environmental remediation.

### 3.25. Modified Pillared Clays

Pillared clays are a form of layered material that has been intercalated with metal oxide pillars to establish a porous and stable structure. This modification increases the thermal stability and surface area of the clay, rendering it a highly effective adsorbent for a variety of pollutants [108]. Aluminum, zirconium, and titanium oxides are frequently employed as pillaring agents, as they are inserted between the clay layers to create persistent porosity. Modified pillared clays exhibit high adsorption capacities for heavy metals, organic pollutants, and radioactive contaminants. The layered structure enables the diffusion of contaminants into the material, while the metal oxide pillars serve as active sites for adsorption. Furthermore, the thermal and chemical stability of pillared clays enables their application in challenging environmental conditions [109].

The effectiveness of modified pillared clays in the removal of a diverse array of contaminants from water and effluent has been demonstrated through research. By integrating various metal oxides or functional groups, these materials can be customized to improve their selectivity for particular pollutants [110]. The applicability of pillared clays in environmental remediation is being further expanded through the development of new pillaring agents and modification techniques.

### 3.26. Modified Zeolites

Zeolites are crystalline aluminosilicates with a highly porous structure, making them effective adsorbents for a wide range of pollutants. Modified zeolites, which have been chemically or physically altered to enhance their adsorption properties, offer even greater potential for environmental applications. Modifications can include ion exchange, impregnation with metal oxides, or functionalization with organic groups [111]. The high surface area and ion exchange capacity of zeolites make them particularly effective for removing heavy metals and ammonium ions from water. Additionally, their porous structure allows for the adsorption of organic pollutants, such as volatile organic compounds (VOCs) and dyes. Modified zeolites can exhibit enhanced selectivity and adsorption capacity, depending on the nature of the modification [112].

Research has shown that modified zeolites are beneficial in various environmental applications, such as groundwater remediation, wastewater treatment, and air purification. The ability to regenerate and reuse zeolites increases their economic viability [113]. Advances in modification techniques continue to broaden the variety of pollutants that zeolites can efficiently remove, making them a versatile and long-lasting solution for environmental remediation.

### 3.27. Molecularly Imprinted Polymers

Molecularly imprinted polymers (MIPs) are synthetic polymers designed to have specific recognition sites for target molecules [114]. These recognition sites are created by polymerizing functional monomers in the presence of a template molecule, which is later removed to leave behind cavities that are complementary in size, shape, and functional groups to the target molecule. This process, known as molecular imprinting, results in highly selective adsorbents. MIPs have gained significant attention for their ability to selectively adsorb specific pollutants from complex mixtures. They are particularly effective for removing trace contaminants, such as pharmaceuticals, pesticides, and endocrine-disrupting chemicals, which are often difficult to remove using conventional adsorbents. The selectivity of MIPs is a major advantage, allowing for the efficient removal of target pollutants without interference from other substances [115].

The application of MIPs in environmental remediation involves their incorporation into various forms, such as beads, membranes, and coatings. These materials have been successfully used to remove a wide range of pollutants from water and wastewater. The development of new imprinting techniques and the use of novel functional monomers continue to enhance the performance and applicability of MIPs in pollutant removal.

### 3.28. Nanocellulose

Nanocellulose is considered a highly promising adsorbent for pollutant removal due to its high surface area, mechanical strength, and biodegradability [116]. Derived from natural sources such as wood pulp, agricultural residues, and certain bacteria, nanocellulose is categorized into three main types: cellulose nanocrystals (CNCs), cellulose nanofibrils (CNFs), and bacterial nanocellulose (BNC). Each type has unique structural characteristics that make it suitable for various adsorption applications [117,118,119].

CNCs and CNFs possess a high density of hydroxyl groups on their surfaces, which can be functionalized to enhance their affinity for specific pollutants [119]. This functionalization can be achieved through various chemical modifications, such as grafting with carboxyl, amine, or sulfonate groups, thereby improving their capacity to adsorb heavy metals, dyes, and organic pollutants. Bacterial nanocellulose, produced by certain strains of bacteria, is known for its high purity and unique three-dimensional network structure, making it particularly effective for use in water filtration membranes [120,121].

Research has shown that nanocellulose-based adsorbents can achieve high removal efficiencies for a variety of contaminants [122]. For example, modified CNCs have shown excellent performance in removing heavy metals like lead and cadmium from aqueous solutions, while CNFs functionalized with cationic polymers have been effective in adsorbing anionic dyes [123]. The ability to regenerate and reuse nanocellulose adsorbents without significant loss of performance further enhances their appeal for practical applications in water and wastewater treatment.

### 3.29. Novel Structured Carbon-Based Materials

Novel structured carbon-based materials, like carbon nanofibers, carbon aerogels, and carbon quantum dots, have recently become of interest due to their potential for removing pollutants. These materials have distinct structural characteristics, including a large surface area, adjustable porosity, and modifiable surfaces with functional groups. These attributes account for their exceptional ability to adsorb substances [124]. Carbon nanofibers are created using electrospinning or chemical vapor deposition methods. These fibers are exceptionally strong and have a large surface area. They are very effective in removing heavy metals, organic pollutants, and radionuclides from water. Carbon aerogels, made through sol–gel techniques and supercritical drying, have low density and high porosity, making them ideal for adsorbing a wide range of pollutants [125].

Carbon quantum dots, which are carbon particles of nanoscale size, possess distinctive optical and electrical characteristics and have demonstrated promise in the elimination of pollutants. These materials can be modified with different groups to increase their ability to selectively adsorb specific substances. Ongoing research is being conducted on innovative carbon-based materials with unique structures. This research aims to discover new ways of synthesizing these materials, develop procedures for functionalizing them, and study their potential applications in environmental remediation.

### 3.30. Plant Biomass

Plant biomass, such as aquatic plants, forestry byproducts, and agricultural wastes, contains abundant biopolymers like cellulose, hemicellulose, and lignin. These can be processed to create powerful adsorbents for removing pollutants from water and wastewater. This sustainable approach not only offers a waste management solution but also supports environmental conservation. Plant biomass can be turned into adsorbents through pyrolysis, chemical activation, and functional group impregnation. These methods improve the surface area, porosity, and adsorption capacity of the resulting materials. Adsorbents made from plant biomass have been highly effective in removing organic contaminants, dyes, and heavy metals from water [126].

Research has shown that plant biomass could be a low-cost and effective adsorbent for environmental applications. Developing adsorbents using renewable resources aligns with the principles of the circular economy and sustainable development. Some current research is focused on improving conversion processes, understanding adsorption mechanisms, and evaluating the long-term effectiveness of these adsorbents. The development and application of advanced non-conventional adsorbents, such as carbon nanotubes, metal–organic frameworks, geopolymers, biochar composites, graphene-based composites, innovative structured carbon-based materials, carbonaceous waste derived from oil refineries, carbon-based aerogels from waste paper, 3D graphene-based adsorbents, carbon xerogels, synthetic hydrogels, adsorbents from stainless steel slag, and industrial sludge, represent significant advancements in the realm of pollutant elimination [127]. These materials possess unique characteristics and mechanisms that offer promise for mitigating environmental pollution by enhancing their efficiency, selectivity, and sustainability.

### 3.31. Super-Chalcogens

Super-chalcogens are a class of materials that include compounds containing large chalcogen atoms, such as sulfur, selenium, and tellurium. These materials have unique electronic and chemical properties that make them highly effective for pollutant removal. Super-chalcogens can form strong bonds with heavy metals and organic pollutants, making them ideal for use in water and wastewater treatment applications. The synthesis of super-chalcogens typically involves the incorporation of chalcogen atoms into various frameworks, such as metal–organic frameworks (MOFs) or covalent organic frameworks (COFs). These structures provide a high surface area and many active sites for adsorption. Additionally, the chalcogen atoms can interact with pollutants through various mechanisms, including covalent bonding, van der Waals forces, and hydrogen bonding [18].

Research has shown that super-chalcogens are highly effective in removing heavy metals such as mercury, lead, and cadmium from water. They also exhibit strong adsorption capabilities for organic pollutants, including dyes and pharmaceuticals. The ability to tailor the properties of super-chalcogens by modifying their chemical composition and structure further enhances their versatility and effectiveness in pollutant removal applications.

### 3.32. Synthetic Hydrogels

Synthetic hydrogels, with their crosslinked polymer networks, are exceptionally powerful in eliminating pollutants. Their capacity to absorb and retain significant volumes of water makes them a standout choice for this purpose. Furthermore, by incorporating specific functional groups and pore structures, we can further enhance their adsorption properties. When it comes to removing organic and ionic contaminants from aqueous solutions, synthetic hydrogels are unmatched in their effectiveness [128]. The synthesis of synthetic hydrogels involves the polymerization of monomers like acrylamide, acrylic acid, and methacrylate in the presence of a crosslinking agent. The resulting hydrogels can be customized with various groups to significantly enhance their affinity for specific contaminants. For instance, hydrogels containing carboxyl or amine groups excel at adsorbing heavy metals, while those with hydrophobic groups are good at capturing organic contaminants [129].

Research has demonstrated that synthetic hydrogels have the potential to achieve high removal efficiencies and rapid adsorption kinetics in water and wastewater treatment. These hydrogels are further practical due to their ability to regenerate and be reused. Current research focuses on developing new hydrogel formulations, optimizing functionalization processes, and exploring new applications for these materials in environmental remediation.

## 4. Pollutants Targeted by Innovative Adsorbents

Pollutants, including heavy metals, organic compounds, and emerging contaminants like pharmaceuticals and pesticides, present significant environmental and health challenges. The effectiveness of innovative adsorbents in mitigating these pollutants is crucial for advancing purification and remediation technologies. Table 2 offers a detailed comparison of the efficiency of various adsorbents in removing specific contaminants, providing valuable insights into their potential applications across different environmental settings. Matching the right adsorbent to a specific pollutant is key to optimizing removal efficiency and minimizing operational costs. Here, we categorize some common pollutants and the adsorbents that have shown the greatest efficacy in removing them (Table 2).

For the removal of pesticides, both magnetic iron-containing carbon and MOFs demonstrate high adsorption efficiency. While magnetic iron-containing carbon is particularly useful for broad-spectrum pesticide removal due to its magnetic separation capability, MOFs are advantageous for selective removal of specific pesticide molecules owing to their customizable pore structures. When targeting antibiotics, both magnetic iron-containing carbon and MOFs show high removal rates, with MOFs offering improved performance due to their high surface area and porosity, making them suitable for more precise antibiotic capture. Similarly, for UV filters, MOFs outperform traditional adsorbents like hydrochars due to their tailored pore sizes, allowing for more efficient removal of these emerging contaminants.

### 4.1. Alkylphenols and Other Surfactants

Alkylphenols and other surfactants are persistent pollutants extensively utilized in detergents and various industrial operations, posing a significant threat to aquatic life and disrupting endocrine systems. In response to this environmental challenge, various adsorbents such as biochar, zeolites, and activated carbon have been employed to mitigate the impact of these pollutants. Moreover, advancements in adsorbent technology, including the utilization of graphene-based materials and cyclodextrin polymers, have significantly enhanced the efficiency of surfactant and alkylphenol removal [137]. These novel adsorbents leverage mechanisms such as hydrogen bonding and hydrophobic interactions, enabling them to achieve substantial adsorption capacities for a wide range of surfactants. Research indicates that these adsorbents exhibit removal efficiencies exceeding 85%, rendering them valuable for the treatment of wastewater from both residential and commercial sources.

### 4.2. Antibiotics

Antibiotic-resistant bacteria are a result of antibiotics present in water sources, originating from pharmaceutical industries, hospital effluents, and agricultural runoff. Tetracycline, ciprofloxacin, and amoxicillin are some of the antibiotics found in water, which can be removed using adsorbents like activated carbon, graphene-based composites, and chitosan-based polymers. These adsorbents have large surface areas and functional groups that enable efficient interactions with antibiotic compounds [138]. Innovative adsorbents including synthetic hydrogels and metal–organic frameworks have been developed with enhanced antibiotic adsorption capacities. These substances have clearance efficiencies of over 90%, reducing antibiotic concentrations to acceptable levels and lowering the risk of antibiotic resistance emergence.

### 4.3. Anti-Inflammatory Drugs

Anti-inflammatory drugs, such as diclofenac and ibuprofen, are frequently found in water sources due to their widespread use and incomplete removal during wastewater treatment. To address this issue, adsorbents such as molecularly imprinted polymers, activated carbon, and biochar have been utilized. Recent advancements in adsorbent technology, including the use of graphene-based materials and ionic-liquid-enhanced adsorbents, have led to improved efficiency and selectivity in removing anti-inflammatory medications [139]. Research has shown that these innovative adsorbents can effectively remove a variety of anti-inflammatory medications, often achieving removal efficiencies of over 80%. Furthermore, these materials’ practical relevance in treating pharmaceutical-contaminated water is enhanced by their ability to be regenerated and reused.

### 4.4. Disinfectants

Chlorine and chloramine are two disinfectants commonly used in water treatment, but they can generate hazardous byproducts such as haloacetic acids and trihalomethanes (THMs). Adsorbents like charcoal, zeolites, and activated carbon have traditionally been utilized to remove these disinfectants along with their byproducts from water. However, recent advancements in adsorbent technology, including synthetic hydrogels and metal–organic frameworks, have significantly improved the efficacy of pollutant removal [140]. Research indicates that these innovative adsorbents are capable of efficiently extracting disinfectants and their byproducts, reducing their concentrations to safe levels. Furthermore, these materials can be regenerated and reused, further enhancing their practical utility in water treatment applications.

### 4.5. Dyes

The dyes used in the textile, leather, and paper industries are significant contributors to water pollution due to their high visibility, toxicity, and resistance to breaking down. To address this issue, adsorbents such as activated carbon, charcoal, and synthetic polymers have traditionally been utilized to remove dyes. Recent advancements in adsorbents, including cyclodextrin polymers, graphene-based composites, and nanocellulose, have enhanced the effectiveness and selectivity of dye removal [141]. These advanced adsorbents use mechanisms like hydrophobic interactions, hydrogen bonding, and electrostatic interactions to target specific herbicides, like atrazine, glyphosate, and dichlorodiphenyltrichloroethane (DDT).

### 4.6. Fluorides

Excessive fluoride in drinking water can be a significant public health risk, especially in areas where groundwater naturally has high fluoride levels. Overconsumption of fluoride can lead to dental and skeletal fluorosis. To address this issue, various adsorbents such as modified clays, activated alumina, and calcium-based compounds have been used to remove fluoride from water. Advanced adsorbents like metal–organic frameworks and hydroxyapatite nanoparticles have shown exceptional capability in fluoride adsorption due to their large surface area and strong attraction to fluoride ions [142]. Studies indicate that these adsorbents can reduce fluoride concentrations to safe levels according to health guidelines. They are suitable for use in household and community water treatment systems as they work effectively across different pH levels and can be regenerated.

### 4.7. Hormones

Hormones are newly discovered pollutants that can interfere with the endocrine systems of people and aquatic life. These pollutants include endocrine-disrupting substances such as testosterone and estrogen. Techniques for removing hormones from water include the use of adsorbents like molecularly imprinted polymers, activated carbon, and zeolites. Advanced adsorbents, such as metal–organic frameworks and carbon–based aerogels, have shown superior adsorption capabilities due to their large surface areas and functionalized surfaces [143]. Studies have demonstrated that these novel adsorbents have a removal efficiency of over 90% in effectively collecting and eliminating hormones [144]. Adsorbents that can target individual hormones through molecular imprinting or functionalization are highly effective for remediating water sources contaminated with hormones.

### 4.8. Metals and Metalloids

Heavy metals, such as lead, mercury, cadmium, and arsenic, are examples of persistent pollutants that pose serious threats to both human health and the environment. These pollutants can originate from runoff from farms, mining operations, and various industrial processes. Novel adsorbents, such as nanocellulose, modified zeolites, and metal–organic frameworks (MOFs), have demonstrated high efficacy in removing these metals from water. These adsorbents possess large surface areas, functional groups, and adjustable porosity, which allow for strong interactions with metal ions through processes such as complexation, adsorption onto active sites, and ion exchange [145].

Research has shown that through appropriate modifications, adsorbents can effectively remove over 90% of heavy metals from contaminated water sources, making them a viable option for treatment. For instance, biochar composites and graphene-based materials have demonstrated high efficiency in adsorbing lead and mercury [146]. This is attributed to their extensive surface areas and their ability to form strong bonds with these metals. Furthermore, the practicality of these adsorbents for large-scale applications is further enhanced by their capacity for regeneration and reusability.

### 4.9. Per- and Polyfluoroalkyl Substances

Per- and polyfluoroalkyl substances (PFASs) are environmental pollutants that persist over time and are used in various industrial applications, such as non-stick coatings and firefighting foams. These compounds pose a risk to human health due to their resistance to degradation and their ability to accumulate in the environment. To mitigate the impact of PFAS, adsorbents such as ion exchange resins and activated carbon have been utilized. The efficacy of PFAS removal has increased due to recent advancements in adsorbent technologies, including metal–organic frameworks and graphene-based compounds [147]. Research indicates that these innovative adsorbents can achieve high removal efficiencies for PFAS, reducing their concentrations to safe levels. The ability to target specific PFAS compounds through functionalization or modification makes these adsorbents highly effective for treating PFAS-contaminated water sources.

### 4.10. Pesticides

Agricultural pesticides often contaminate water bodies through leaching and runoff, posing risks to human health and aquatic life. Adsorbents such as activated carbon, modified clays, and biochar composites have been utilized to remove pesticides from water [148]. Recent advancements in adsorbent materials, including molecularly imprinted polymers (MIPs) and metal–organic frameworks (MOFs), have significantly enhanced the capacity to effectively and selectively remove pesticides. These advanced adsorbents can target specific pesticides, including atrazine, glyphosate, and dichlorodiphenyltrichloroethane (DDT), through mechanisms like hydrophobic interactions, hydrogen bonding, and electrostatic interactions. Research has demonstrated that these adsorbents can achieve high removal efficiencies, making them suitable for treating agricultural runoff and contaminated groundwater [148].

### 4.11. Plastic Nanoparticles

Plastic nanoparticles represent a novel form of pollutants that have the potential to accumulate in water bodies, posing threats to both aquatic life and human health. These particles are generated because of the breakdown of larger plastic waste. To mitigate the impact of plastic nanoparticles, various adsorbents including activated carbon, biochar, and modified clays have been utilized. Advancements in adsorbent materials, particularly those involving composites based on graphene and polymers imprinted with molecules, have led to enhanced efficacy in removing plastic nanoparticles [149]. These advanced adsorbents, functioning through mechanisms such as hydrophobic interactions and electrostatic attraction, have demonstrated significant capabilities in adsorbing a wide range of plastic nanoparticles. Research indicates that these innovative adsorbents are capable of achieving up to 90% removal efficiency, thereby offering promise in the purification of water contaminated with plastic nanoparticles [150]. Innovative adsorbents have shown significant promise in the removal of various contaminants from water, including radionuclides, fluorides, dyes, pesticides, antibiotics, anti-inflammatory medications, hormones, synthetic ingredients for cosmetics, UV filters, alkylphenols, polycyclic aromatic hydrocarbons, per- and polyfluoroalkyl compounds, and plastic nanoparticles. These materials possess unique characteristics and operational processes that enhance their sustainability, efficiency, and selectivity. Consequently, they offer promising alternatives for addressing environmental pollution.

### 4.12. Polycyclic Aromatic Hydrocarbons

Pollutants often found in soil and water that are hazardous and carcinogenic are referred to as polycyclic aromatic hydrocarbons, or PAHs. They are formed during the incomplete combustion of organic matter. Adsorbents such as zeolites, activated carbon, and biochar have been used to remove PAHs. Advanced adsorbents, including metal–organic frameworks and graphene–based composites, have demonstrated superior adsorption capabilities for PAHs [151]. These innovative adsorbents, which operate through π-π interactions and hydrophobic interactions, have shown the ability to remove PAHs with high efficiency, often exceeding 90%. Furthermore, their practical relevance in treating soil and water contaminated with PAHs is heightened by their capacity for regeneration and reusability [152].

### 4.13. Radionuclides

Radionuclides such as uranium, cesium, and strontium pose a significant risk due to their radioactive properties and extended periods of decay. This results in the long-lasting contamination of water sources, especially in close proximity to nuclear facilities and areas impacted by nuclear incidents. New types of adsorbents have been created to specifically target radionuclides. These include hydroxyapatite nanoparticles, iron-based hybrid nanomaterials, and modified zeolites. These materials utilize various mechanisms to efficiently capture and immobilize radionuclides, including ion exchange, surface complexation, and precipitation [153].

Research has demonstrated that certain adsorbents, such as hydroxyapatite, possess the remarkable ability to selectively eliminate radionuclides, even when faced with the challenge of competing ions. This results in exceptionally high removal efficiencies. These materials show great potential for effectively removing radioactive contamination from both groundwater and surface water due to their impressive stability and selectivity.

### 4.14. Synthetic Cosmetics Ingredients

Wastewater discharges frequently introduce synthetic compounds found in cosmetics, such as phthalates and parabens, into bodies of water. Both human health and aquatic life may be harmed by these substances. To mitigate the impact of these pollutants, various adsorbents including modified clays, activated carbon, and biochar have been utilized. Advancements in adsorbent materials, such as graphene-based composites and cyclodextrin polymers, have significantly enhanced the efficiency of removing synthetic chemicals from cosmetics [154]. These advanced adsorbents can adsorb a wide range of synthetic substances with high capacities, owing to mechanisms such as electrostatic attraction, hydrogen bonding, and hydrophobic interactions. Studies have demonstrated that innovative adsorbents can achieve removal efficiencies of over 85%, making them valuable for treating wastewater from the cosmetic industries.

### 4.15. UV Filters

UV filters are emerging pollutants that can accumulate in water bodies and pose a threat to aquatic ecosystems. These compounds are commonly present in sunscreens and various personal care products. Efforts to mitigate their impact involve treating water contaminated with UV filters, such as octinoxate and oxybenzone, using adsorbents like activated carbon, charcoal, and modified clays. The effectiveness of UV filter removal has been significantly enhanced through advancements in adsorbent materials, including graphene-based composites and molecularly imprinted polymers [155]. The utilization of these advanced adsorbents allows for high adsorption capacities for a variety of UV filters, owing to mechanisms such as π-π stacking and hydrophobic interactions. Studies have demonstrated that these innovative adsorbents can achieve removal efficiencies of over 90%, making them invaluable for the treatment of wastewater containing UV filters.

## 5. Adsorbent Performance and Efficiency

### 5.1. Adsorption Capacity

One of the most critical factors in determining the efficacy of an adsorbent in removing contaminants from water is its adsorption capacity. This capacity refers to the maximum concentration of a specific contaminant that an adsorbent can absorb per unit mass or volume. Various parameters, including surface area, pore size distribution, and the presence of functional groups on the adsorbent, influence the adsorption capacity. Greater surface areas and optimal pore shapes often provide more active sites for pollutant interaction, thereby enhancing the adsorption capabilities [156]. Due to their extensive surface areas and varying porosities, advanced adsorbents such as modified zeolites, graphene-based composites, and metal–organic frameworks (MOFs) have demonstrated remarkably high adsorption capacities. MOFs, for instance, have exhibited adsorption capabilities of hundreds of milligrams per gram for organic contaminants and heavy metals. Similarly, graphene-based composites, owing to their strong π-π interactions and hydrogen bonding capacities, have shown potential in effectively removing significant quantities of dyes and medications [157].

Recent studies on sustainable cellulose derivatives have also demonstrated promising adsorption capacities, particularly for organic pollutants such as dyes and pharmaceuticals [43,158]. While their adsorption capacity may be moderate compared to advanced materials like MOFs or graphene-based composites, cellulose derivatives offer a distinct advantage in terms of sustainability. These materials exhibit a pseudo-second-order kinetic behavior, indicating chemisorption as the dominant mechanism. Additionally, thermodynamic analysis reveals that their adsorption processes are spontaneous and endothermic, similar to other bio-based adsorbents like biochar and nanocellulose. Furthermore, their renewable nature and ability to regenerate with minimal efficiency loss make them attractive for large-scale, cost-effective water treatment applications.

The adsorption capacity of different adsorbents is often determined using adsorption isotherms, such as the Freundlich and Langmuir models. These models assist in the understanding of adsorption behavior and in the development of materials with ideal characteristics for specific pollutants. Current research aims to enhance the adsorption capability of innovative adsorbents through the manipulation of their surface chemistry and structural properties.

### 5.2. Selectivity

Selectivity plays a pivotal role in characterizing an adsorbent’s capacity to preferentially adsorb specific contaminants from a mixture of different pollutants. The significance of high selectivity is especially pronounced in applications where the desired pollutant exists in low concentrations among a diverse range of competing substances. Selectivity hinges on the chemical affinity between the adsorbent and the adsorbate, as well as the congruence of the adsorbent’s pore size and shape with the target molecules [159]. Molecularly imprinted polymers (MIPs) represent a notable instance of highly selective adsorbents. These polymers are created using template molecules that generate particular binding sites customized to the target pollutants, enabling selective adsorption even in intricate matrices. Cyclodextrin polymers and specific functionalized MOFs also demonstrate high selectivity owing to their capacity to form inclusion complexes or coordinate with specific contaminants [160].

Improving the selectivity of adsorbents frequently includes modifying their surfaces with specific groups that exhibit strong affinities for the target pollutants. For instance, thiol-functionalized adsorbents demonstrate high selectivity for mercury ions, whereas amine-functionalized materials display a preference for acidic pollutants. The advancement of adsorbents with high selectivity is essential for applications in wastewater treatment, particularly in scenarios characterized by diverse and complex contaminant profiles.

### 5.3. Regeneration and Reusability

Regeneration and reusability are crucial factors in determining the long-term viability and cost-effectiveness of the adsorption process. The capability of an adsorbent to be regenerated and reused multiple times without substantial loss of performance leads to reduced overall operational costs and minimized environmental impact [161]. Typically, regeneration involves desorbing the adsorbed pollutants using techniques such as thermal treatment, solvent washing, or pH adjustment. Numerous innovative adsorbents, such as activated carbon, biochar, and specific MOFs, have exhibited excellent regeneration and reusability. For example, activated carbon can be thermally regenerated at high temperatures, while biochar can be regenerated through simple washing procedures. Owing to their robust framework structures, MOFs can endure multiple regeneration cycles without significant degradation [162].

The efficiency of regeneration processes relies on the characteristics of the adsorbent and the nature of the pollutants. Ongoing research is dedicated to developing energy-efficient and environmentally friendly regeneration methods. For instance, the use of mild solvents or natural acids for regeneration can lower the environmental impact and operational costs associated with the adsorption process.

### 5.4. Cost-Effectiveness

An important consideration in the practical application of adsorbents for pollution removal is cost-effectiveness. The costs associated with the production, deployment, regeneration, and disposal of an adsorbent significantly impact its economic feasibility. When compared to synthetic materials, adsorbents derived from abundant and renewable resources, such as industrial byproducts, natural minerals, and agricultural residues, are generally more cost-effective [163]. Innovative adsorbents, characterized by low raw material costs and simple production methods, exemplify substantial economic advantages. For example, biochar, geopolymers, and modified clays are promising adsorbents due to their inexpensive raw materials and straightforward production processes. Biochar, derived from various biomass sources through pyrolysis, can be prominently scaled up using current technologies. Similarly, geopolymers, fashioned from industrial waste products like slag and fly ash, offer a cost-effective alternative to traditional adsorbents [164].

The cost-effectiveness of an adsorbent is also contingent upon its adsorption capacity, selectivity, and regeneration efficiency. Adsorbents with high performance and reusability diminish the need for frequent replacement and disposal, ultimately reducing operational costs. Therefore, the development of adsorbents that effectively balance high performance with low production costs is crucial for their widespread adoption in water and wastewater treatment applications.

### 5.5. Environmental Impact

The environmental impact of adsorbents spans their entire lifecycle, from production and usage to regeneration and disposal. Adsorbents derived from sustainable sources, such as plant biomass and agricultural residues, or those incorporating industrial byproducts, contribute to sustainability by valorizing waste and reducing reliance on new materials. Moreover, the production processes of these adsorbents should prioritize energy efficiency and minimize waste generation. Innovative adsorbents like biochar, plant biomass, and geopolymers exhibit relatively modest environmental footprints due to their sustainable origins and environmentally conscious production methods. Furthermore, adsorbents capable of multiple regenerations and reuses help mitigate solid waste generation and lessen the environmental impact associated with frequent replacements [128]. Another environmental concern is the disposal of exhausted adsorbents. A circular economy is facilitated by adsorbents that can be securely disposed of or repurposed at the conclusion of their lifecycle. For instance, discarded biochar can be utilized as a soil amendment, which not only provides agricultural benefits but also sequesters contaminants. Sustainable water and wastewater treatment solutions necessitate the development of adsorbents that have a minimal environmental impact throughout their lifecycle [164].

### 5.6. Comparative Performance of Adsorbents for Selected Pollutants

Adsorption performance varies significantly depending on the type of adsorbent and pollutant, as well as the experimental conditions such as pH, contact time, and adsorbent dose. Table 3 presents a comparison of key adsorbents used for the removal of antibiotics (amoxicillin), dyes (Methylene Blue), pesticides, and fluoride. Metal–organic frameworks (MOFs) show high adsorption capacities for amoxicillin, particularly under mildly acidic conditions (pH 6.8). Similarly, cellulose-based hydrogels exhibit substantial adsorption capacity for Methylene Blue, making them highly effective for dye removal in neutral conditions (pH 7.0). For pesticide removal, magnetic iron-containing carbon demonstrates effective adsorption at pH 6.5, while hydroxyapatite nanoparticles exhibit good fluoride removal capacities in slightly acidic environments (pH 6.2). These results highlight the importance of selecting the appropriate adsorbent based on the specific pollutant and operating conditions to achieve optimal adsorption performance.

## 6. Case Studies and Applications

### 6.1. Industrial Applications

Adsorbents are utilized in various industries such as mining, food processing, manufacturing, and pharmaceuticals to eliminate pollutants. One well-known example is the use of activated carbon in the food and beverage industry to remove color and impurities from products like juices, wine, and sugar. By adsorbing unwanted colorants, odors, and contaminants, activated carbon ensures the safety and quality of the product. In the pharmaceutical industry, activated carbon is also used to enhance the quality of pharmaceuticals by removing organic impurities and residual solvents [169]. In the mining sector, acid mine drainage (AMD), which contains high concentrations of heavy metals and acidity, is treated using new adsorbents such as biochar and modified zeolites. These adsorbents efficiently remove metals like arsenic, lead, and cadmium, reducing the negative environmental impact of mining. Geopolymers, derived from industrial waste materials like fly ash, have also been used to clean mine water, showing excellent results in metal adsorption and acidity neutralization [170].

The use of advanced adsorbents such as graphene-based composites and cyclodextrin polymers is advantageous for the textile sector, which is known for its high water consumption and discharge of dye-laden effluents. These substances help remove a variety of colors from wastewater, enabling water reusability and reducing pollution levels in the environment. Incorporating these adsorbents into existing treatment systems can enhance the overall effectiveness and sustainability of managing industrial wastewater.

### 6.2. Pilot Studies

Pilot studies are crucial for demonstrating the effectiveness and practicality of new adsorbents. These studies bridge the gap between laboratory research and real-world industrial applications, providing valuable insights into adsorbent performance and operating parameters. For instance, pilot studies have been conducted to evaluate the use of biochar in municipal wastewater treatment plants [171]. Research suggests that biochar could be a promising alternative for enhancing current treatment methods by significantly reducing concentrations of nutrients, heavy metals, and medications. Using molecularly imprinted polymers (MIPs) to selectively remove endocrine-disrupting chemicals (EDCs) from drinking water is another example [115]. Even in the presence of competing drugs, MIPs have been shown in pilot-scale trials to obtain excellent removal efficiency for molecules, such as bisphenol A (BPA) and estradiol. This research demonstrates the potential of MIPs to improve the standard and safety of drinking water sources. Metal–organic frameworks (MOFs) have been studied for use in air filtration systems [172]. Pilot investigations have shown that MOFs are highly effective at absorbing volatile organic compounds (VOCs) and other airborne contaminants due to their large surface area and adjustable pore architectures. This makes MOFs a promising solution for reducing indoor air pollution and promoting a cleaner, healthier work environment. Pilot installations in industrial buildings have demonstrated the feasibility of using MOFs for this purpose.

### 6.3. Field Trials

In order to validate the durability and long-term performance of adsorbents in real environmental conditions, field trials are necessary. These trials involve placing adsorbents in both artificial and natural settings such as constructed wetlands, contaminated water bodies, or large-scale treatment facilities to assess their effectiveness over time. An excellent example of this is the use of hybrid nanomaterials based on iron for groundwater remediation. Field tests have shown that these substances can effectively remove heavy metals such as arsenic from compromised aquifers, providing a sustainable method for improving water quality in affected areas [173]. The use of carbon-based aerogels made from waste paper for cleaning up oil spills is the focus of another significant field experiment. These aerogels have been utilized in maritime environments to soak up oil spills and have shown their ability to effectively remove hydrocarbons from water surfaces. Their lightweight and hydrophobic properties make them ideal for emergencies that necessitate rapid deployment and efficient oil recovery [172].

Additionally, field tests have been carried out to assess the efficacy of adsorbents produced from plant biomass in the treatment of agricultural runoff. These adsorbents, which are made of materials like coconut shells and rice husks, have been tried in buffer strips and artificial wetlands to extract pesticides and nutrients from agricultural runoff. Significant decreases in pollutant loads were observed in the results, suggesting that plant biomass adsorbents may be useful for reducing the negative effects of agriculture on water quality.

## 7. Challenges and Future Perspectives

### 7.1. Technical Challenges

Despite the significant progress in developing novel adsorbents, several technological challenges remain. One of the primary obstacles is the scalability of synthesis procedures. Many advanced adsorbents, such as graphene-based materials and metal–organic frameworks (MOFs), are produced through complex and costly processes that are difficult to scale up to industrial levels. To achieve widespread adoption of these materials, it is crucial to develop scalable and cost-effective production methods. Another significant challenge is ensuring the stability and longevity of adsorbents in real-world applications. Adsorbents must maintain their functionality despite fluctuations in temperature, pH, and the presence of competing ions. Although materials like MOFs and nanocomposites tend to be resilient, others, such as activated carbon and biochar, may deteriorate or become less effective over time. To enable their sustained use in environmental cleanup, it is crucial to improve the durability and stability of these adsorbents [174].

Adsorbent regeneration and reuse present additional technical challenges. Effective regeneration methods must be developed to restore the adsorption capacity of spent adsorbents without causing significant material loss or degradation. While some adsorbents can be easily recycled through simple techniques like washing or heating, others require more complex and costly procedures. Developing efficient and sustainable regeneration methods is crucial to ensuring the viability of adsorbent-based pollutant removal systems.

### 7.2. Economic Considerations

Economic factors play a significant role in the adoption and application of novel adsorbents. The costs of synthesis, processing, and raw materials can be substantial barriers to commercialization. For example, while advanced adsorbents like MOFs and graphene-based composites are often prohibitively expensive for large-scale use, materials such as activated carbon and biochar are more affordable [175]. To make these advanced adsorbents more commercially viable, it is essential to reduce production costs through economies of scale, the use of less expensive raw materials, and improved synthesis techniques. Economic considerations also encompass the costs of regenerating and disposing of spent adsorbents. Adsorbents that can be regenerated easily and inexpensively are more advantageous from a financial perspective. However, for those that cannot be regenerated, disposal costs and potential environmental impacts must be factored in. Developing materials that strike a balance between high performance, low production costs, and ease of regeneration is crucial for the economic viability of adsorbents [176].

The efficiency and lifespan of adsorbents directly affect the overall cost-effectiveness of their use in pollutant removal. Adsorbents with higher adsorption capacities and longer lifespans require fewer replacements and less maintenance, leading to lower operating costs. To fully evaluate the costs and benefits of using adsorbent-based technologies across different environmental applications, economic assessments and lifecycle analyses are essential.

### 7.3. Regulatory and Policy Aspects

The adoption of innovative adsorbents for pollutant removal is significantly shaped by policy and regulatory frameworks. Regulatory standards for air and water quality establish permissible limits for various contaminants, driving the need for effective treatment methods. For adsorbents to be considered viable for environmental remediation, they must meet these regulatory requirements. Nevertheless, the regulatory approval process can be time-consuming and complex, especially for new materials and technologies. Ensuring that innovative adsorbents comply with environmental, health, and safety regulations is essential for their commercialization. This involves conducting thorough risk assessments and testing to prove their safety and effectiveness across different settings [175,176].

Policy incentives and support are also key in promoting the use of advanced adsorbents. Government initiatives that encourage research and development, fund pilot projects, and offer tax incentives for environmentally friendly technologies can significantly accelerate the adoption of these innovations. Collaboration between industry, academia, and government agencies is crucial for aligning research efforts with regulatory and policy objectives.

### 7.4. Future Research Directions

Future research on adsorbents for pollutant removal should focus on addressing current challenges and exploring new opportunities. Key areas include developing scalable, cost-effective synthesis methods and incorporating green chemistry principles to use renewable resources and environmentally friendly processes. This approach can lower production costs and environmental impact. Additionally, exploring new materials and improving stability, regeneration, and long-term durability will enhance the effectiveness and sustainability of adsorbents.

An additional key area of research should be designing adsorbents with enhanced selectivity and stability. This involves developing multifunctional adsorbents capable of targeting multiple contaminants at once while maintaining effectiveness under diverse environmental conditions. Advances in materials science, including nanotechnology and hybrid materials, could greatly enhance the properties and performance of these adsorbents.

Research should also emphasize the regeneration and reuse of adsorbents, which is critical for their practical application. Effective and sustainable regeneration techniques are necessary to ensure that adsorbents can be used multiple times without significant loss of performance. This involves exploring advanced regeneration methods, such as utilizing renewable energy sources like solar or wind power to drive regeneration processes, or employing biodegradable solvents that are less harmful to the environment. Such approaches can help reduce both the environmental impact and the operational costs associated with regenerating adsorbents, making them more viable for long-term use in various applications. Additionally, developing methods that improve the efficiency and ease of regeneration can further enhance the overall practicality and sustainability of adsorbent technologies.

Innovation in adsorbent technology depends heavily on collaboration and interdisciplinary research. Effective solutions to both technical and regulatory challenges are best developed through partnerships among materials scientists, chemists, environmental engineers, and policymakers. Additionally, prioritizing field trials and real-world applications is crucial for verifying the efficacy of new adsorbents and gaining practical insights into their implementation.

## 8. Conclusions

Recent years have seen significant advancements in the development and application of innovative adsorbents for removing pollutants from water and wastewater. Driven by the need for more efficient, cost-effective, and environmentally friendly solutions to combat increasing water pollution, these advancements highlight the progress in this field. This review covers a diverse array of adsorbents, including metal–organic frameworks (MOFs), graphene-based composites, biochar, and molecularly imprinted polymers (MIPs). These materials demonstrate notable versatility and potential for addressing a wide range of contaminants.

Numerous studies have demonstrated that these innovative adsorbents exhibit exceptional adsorption capacities for a broad spectrum of pollutants, including heavy metals, radionuclides, fluorides, dyes, pesticides, antibiotics, and other emerging contaminants. Their high surface area, adjustable pore structures, and customizable surfaces enable strong interactions with target pollutants, leading to high removal efficiencies. Additionally, the ability to regenerate and reuse many of these adsorbents enhances their practicality and cost-effectiveness, making them viable options for large-scale water and wastewater treatment.

Implementing these adsorbents presents several challenges. Key technical issues include scaling up synthesis processes, ensuring stability under real-world conditions, and developing effective regeneration techniques. Addressing these issues is crucial for maximizing the potential of these materials. Economic factors also play a significant role; the costs associated with raw materials, synthesis, and regeneration impact the feasibility of these adsorbents for industrial applications. Additionally, regulatory and policy frameworks must support the use of these advanced materials by ensuring they meet safety and environmental standards and by providing incentives for their development and deployment.

Future research holds broad implications. Continued development is needed to establish scalable and cost-effective synthesis methods for producing high-performance adsorbents on an industrial scale. Additionally, research should prioritize enhancing adsorbent selectivity and stability to effectively target specific pollutants within complex environmental contexts. This involves creating multifunctional adsorbents that can address multiple contaminants simultaneously, thus boosting the overall efficiency of water treatment processes.

Developing sustainable regeneration techniques is a critical area for future research. Implementing environmentally friendly and efficient methods for regenerating spent adsorbents will reduce operational costs and minimize waste, thereby enhancing the sustainability of adsorption-based treatment systems. Furthermore, fostering innovation and ensuring that new developments align with regulatory and policy objectives will require interdisciplinary collaboration among materials scientists, environmental engineers, chemists, and policymakers.

Prioritizing field trials and real-world applications is essential for validating the performance of new adsorbents under practical conditions. Such studies provide valuable insights into operational parameters, long-term durability, and overall effectiveness across different environmental contexts. These data are vital for transitioning from laboratory research to large-scale industrial applications, facilitating the move from innovative concepts to practical solutions.

Overall, the progress in developing innovative adsorbents marks a significant advancement in addressing the global issue of water pollution. These materials offer promising solutions that combine high efficiency, cost-effectiveness, and environmental sustainability. By overcoming existing technical and economic challenges and establishing supportive regulatory and policy frameworks, these advanced adsorbents could transform water and wastewater treatment practices. Continued research and collaboration will be crucial to fully harness their potential and ensure the provision of safe, clean water for future generations.

## Figures and Tables

**Table 1:** Adsorbents, their mechanisms, and target pollutants in different applications.

Adsorbent	Adsorption Mechanism	Application	Advantages	Disadvantages
Activated carbon	Physisorption	Organic compounds, dyes, VOCs	High surface area, cost-effective	Limited capacity for heavy metals
Biochar	Physisorption	Organic pollutants, nutrients	Renewable, cost-effective	Limited selectivity
Calixarene-based polymers	Chemisorption	Metals, organic compounds	High selectivity	High synthesis cost
Carbon nanotubes (CNTs)	Physisorption	Organic pollutants, gases	Large surface area, mechanical strength	High cost, toxicity concerns
Chitosan-based nanocomposites	Chemisorption	Metals, dyes	Biodegradable, selective	Lower capacity than activated carbon
Cyclodextrin polymers	Chemisorption	Pharmaceuticals, organic pollutants	Selective binding for small molecules	Limited reusability
Geopolymers	Chemisorption	Metals, ammonium	High ion exchange capacity	Fragile under acidic conditions
Graphene-based composites	Physisorption/chemisorption	Organic compounds, metals	High surface area, tunable properties	Expensive, hard to scale
Hydroxyapatite nanoparticles	Chemisorption	Metals, radionuclides	High affinity for heavy metals	Lower adsorption for organics
Metal–organic frameworks (MOFs)	Chemisorption	Metals, gases	High selectivity, large surface area	Expensive, less thermally stable
Nanocellulose	Physisorption	Dyes, pharmaceuticals	Renewable, biodegradable	Limited selectivity for metals
Zeolites	Chemisorption	Metals, ammonium	High ion exchange capacity	Expensive modification required

**Table 2:** Efficiency of adsorbents in removing specific pollutants.

Pollutant Category	Effective Adsorbents	Efficiency/Observations	Reference
Alkylphenols and other surfactants	Hydrochars from sewage sludge	Efficient in adsorbing alkylphenols and surfactants.	[130]
Antibiotics	Magnetic iron-containing carbon	High removal efficiency for antibiotics.	[131]
Anti-inflammatory drugs	Hydrochars from sewage sludge	Effective in adsorbing anti-inflammatory drugs from wastewater.	[130]
Disinfectants	Hydrochars from sewage sludge	High efficiency in adsorbing disinfectants.	[130]
Dyes	CMC hydrogel, magnetite/carbon nanocomposites, clays, activated charcoal	CMC hydrogel >90% for Methylene Blue; Magnetite/carbon shows high capacities for various dyes; Clays and charcoal also efficient for dye removal.	[132,133]
Fluorides	Magnetic iron-containing carbon	Effective removal of fluorides from water.	[131]
Hormones	Hydrochars from sewage sludge	Efficient in removing hormones from wastewater.	[130]
Metals and metalloids	Washingtonia Robusta, magnetic iron-containing carbon, CNTs, biochar	Efficient removal of Fe, Ni, Cu, Cr, Pb, Co, and Al from wastewater. Magnetic adsorbents effective across a wide range of metals. Biochar and CNTs also show high efficiency.	[131,134]
Per- and polyfluoroalkyl substances (PFASs)	Activated charcoal	Efficient in removing PFAS from water.	[135]
Pesticides	Magnetic iron-containing carbon	Effective in adsorbing various pesticides.	[131]
Plastic nanoparticles	Hydrochars from sewage sludge	Effective in removing plastic nanoparticles from wastewater.	[130]
Polycyclic aromatic hydrocarbons (PAHs)	Soil-based filter with zeolite	Increased removal rates of PAHs with enhanced filter media using zeolite.	[136]
Radionuclides	Magnetic iron-containing carbon	High efficiency in removing radionuclides from aquatic media.	[131]
Synthetic cosmetic ingredients	Hydrochars from sewage sludge	Efficient in removing synthetic cosmetic ingredients from effluent.	[130]
UV filters	Hydrochars from sewage sludge	Effective removal of UV filters from wastewater.	[130]

This table provides an overview of the most effective adsorbents for each specific pollutant category.

**Table 3:** Adsorption performance of selected adsorbents for various pollutants.

Pollutant	Adsorbent	Adsorption Capacity (mg/g)	pH	Contact Time (h)	Adsorbent Dose (g/L)	References
Amoxicillin (antibiotic)	Metal–organic frameworks (MOFs)	320.0	6.8	5.0	0.2	[165]
Methylene Blue (Dye)	Cellulose-based hydrogels	450.0	7.0	2.5	0.6	[166]
Pesticides	Magnetic iron-containing carbon	120.0	6.5	6.0	0.5	[167]
Fluoride	Hydroxyapatite nanoparticles	95.4	6.2	6.0	1.0	[168]
